# Comparative Evaluation and Optimization of Auxin Type and Concentration on Rooting Efficiency of *Photinia* × *fraseri* Dress: Stem Cuttings Using Response Surface Methodology

**DOI:** 10.3390/plants14152420

**Published:** 2025-08-04

**Authors:** Gülcay Ercan Oğuztürk, Müberra Pulatkan, Cem Alparslan, Türker Oğuztürk

**Affiliations:** 1 Department of Landscape Architecture, Recep Tayyip Erdoğan University, Rize 53020, Türkiye; gulcay.ercanoguzturk@erdogan.edu.tr; 2 Department of Landscape Architecture, Karadeniz Technical University, Trabzon 61080, Türkiye; muberra@ktu.edu.tr; 3 Department of Mechanical Engineering, Recep Tayyip Erdoğan University, Rize 53020, Türkiye; cem.alparslan@erdogan.edu.tr

**Keywords:** *Photinia × fraseri* Dress., IBA, response surface methodology, rooting percentage, vegetative propagation, stem cuttings

## Abstract

This study aimed to evaluate and optimize the effects of three auxin types—indole-3-butyric acid (IBA), naphthaleneacetic acid (NAA), and indole-3-acetic acid (IAA)—applied at four concentrations (1000, 3000, 5000, and 8000 ppm) on the rooting performance of *Photinia × fraseri* Dress. stem cuttings. The experiment was conducted under controlled greenhouse conditions using a sterile perlite medium. Rooting trays were placed on bottom-heated propagation benches maintained at a set temperature of 25 ± 2 °C to stimulate root formation. However, the actual rooting medium temperature—measured manually every four days from the perlite zone using a calibrated thermometer—ranged between 18 °C and 22 °C, with an overall average of approximately 20 ± 2 °C. The average values of these root-zone temperatures were used in the statistical analyses. Rooting percentage, root number, root length, callus formation, and mortality rate were recorded after 120 days. In addition to classical one-way ANOVA, response surface methodology (RSM) was employed to model and optimize the interactions between auxin type, concentration, and temperature. The results revealed that 5000 ppm IBA significantly enhanced rooting performance, yielding the highest rooting percentage (85%), average root number (5.80), and root length (6.30 cm). RSM-based regression models demonstrated strong predictive power, with the model for rooting percentage explaining up to 92.79% of the total variance. Temperature and auxin concentration were identified as the most influential linear factors, while second-order and interaction terms—particularly T·ppm—contributed substantially to root length variation. These findings validate IBA as the most effective exogenous auxin for the vegetative propagation of *Photinia × fraseri* Dress. and provide practical recommendations for optimizing hormone treatments. Moreover, the study offers a robust statistical modeling framework that can be applied to similar propagation systems in woody ornamental plants.

## 1. Introduction

The global demand for ornamental plants continues to rise in parallel with the rapid expansion of urban landscapes and the growing emphasis on sustainable green infrastructure. Ornamental shrubs are integral to urban planning projects due to their aesthetic appeal [1], contributions to microclimate regulation, and soil stabilization capabilities [2,3]. Among these, *Photinia × fraseri* Dress., a hybrid evergreen shrub belonging to the Rosaceae family, stands out for its dense growth habit, striking red-bronze new foliage, and adaptability to diverse climatic and edaphic conditions. It is frequently used in hedges, mass plantings, urban borders, and visual screens, making it a staple in landscape architecture across temperate and subtropical regions [4,5,6].

In Turkey and several Mediterranean countries, *Photinia × fraseri* has gained popularity in both public and private green spaces due to its high ornamental appeal and relatively low maintenance requirements [7]. Its evergreen nature ensures year-round visual continuity in designed landscapes, and its tolerance to pollution, pruning, and drought makes it an ecologically resilient choice for urban environments [8,9]. Studies have shown that *Photinia* species exhibit adaptive mechanisms to abiotic stressors, such as temperature fluctuations, salinity, and drought, key factors in climate-resilient plant selection [10,11].

Despite its landscape popularity, large-scale propagation of *Photinia × fraseri* remains limited by the inefficiencies of seed-based propagation, which often results in genetic variability, low germination rates, and inconsistent growth [12]. Vegetative propagation via stem cuttings offers a more reliable alternative, ensuring genetic uniformity and rapid multiplication of elite genotypes [13,14]. Semi-hardwood stem cuttings are commonly preferred in ornamental propagation due to their practicality and higher rooting success under controlled conditions [2,3,6]. A key determinant of vegetative propagation success is the application of auxins—plant growth regulators that stimulate cell division and root primordium initiation [15]. Among them, indole-3-butyric acid (IBA) is widely recognized for its superior effectiveness, attributed to its chemical stability and resistance to enzymatic degradation [16,17]. Naphthaleneacetic acid (NAA), while effective in some species, may induce excessive callus formation or phytotoxic effects at high doses [14,18]. Indole-3-acetic acid (IAA), though naturally occurring in plants, is typically less effective in exogenous applications due to its instability and rapid breakdown [19,20,21,22]. Auxin effectiveness is also concentration-dependent, typically exhibiting a biphasic response: low to moderate concentrations promote rooting, while excessive levels may inhibit root elongation or induce necrosis [14,18,23,24]. For example, Bowden et al. [13] found 1000–3000 ppm IBA optimal for lavender and rose cuttings, whereas higher doses negatively affected root formation. Similarly, Nazari et al. [25] demonstrated that 3000–5000 ppm IBA improved rooting in *Thymus vulgaris*, with variation depending on plant sex and species. However, data on *Photinia × fraseri* remain scarce and fragmented. Ersoy [7] reported successful rooting at 3000 ppm IBA, yet no consensus has been reached on optimal dose ranges for other auxins or combined treatments. Beyond hormonal factors, rooting success depends on a complex interaction with environmental conditions, such as temperature, humidity, substrate composition, and light quality [20,21,22]. For example, Katsanou et al. [26] reported that combining red LED light with IBA maximized in vitro rooting in *Cistus creticus*, indicating a synergy between light spectrum and hormonal cues.

Recent advances in plant biotechnology emphasize the role of biological agents, such as plant growth-promoting rhizobacteria (PGPR), *Trichoderma* spp. and endophytic fungi like *Piriformospora indica*, which enhance root formation by producing natural auxins and improving nutrient uptake and water retention. These microorganisms are increasingly viewed as sustainable complements or alternatives to synthetic auxins in vegetative propagation systems [10,11,23,24,27,28]. To evaluate such multifactorial interactions—auxin type, concentration, and environmental conditions—statistically robust modeling techniques are required. One such method is response surface methodology (RSM), which enables the analysis of multiple independent variables and their interaction effects on response parameters, while identifying optimal treatment combinations [29,30,31,32]. By using second-order (quadratic) regression models, RSM efficiently captures both linear and nonlinear responses, reduces the number of experimental trials, and offers high-resolution insights into complex biological systems. In propagation studies, this approach has been increasingly applied to fine-tune hormone dosages and environmental inputs for maximum rooting performance, particularly in ornamental species [33,34,35,36,37].

Therefore, the aim of this study is to investigate the effects of three auxin types (IBA, NAA, and IAA) applied at four different concentrations (1000, 3000, 5000, and 8000 ppm) on the rooting performance of *Photinia × fraseri* Dress. stem cuttings. Using RSM, this study models the influence of these variables on key response parameters, such as rooting percentage, root number, and root length. The resulting models are validated against experimental data, and optimal hormone types and concentrations are identified to support efficient propagation protocols. Unlike previous studies that evaluated auxin effects in isolation, this research integrates hormone type, concentration, and environmental parameters using a response surface approach, offering a comprehensive and predictive framework for optimizing *Photinia × fraseri* propagation.

## 2. Materials and Methods

### 2.1. Plant Material and Cutting Preparation

The cuttings of *Photinia × fraseri* ‘Red Robin’, an evergreen ornamental shrub widely used in urban landscape applications, were obtained from healthy donor plants during the first week of June. Semi-softwood terminal cuttings measuring 10–12 cm in length and containing 4–5 nodes were selected for uniformity in vigor, age, and morphological structure. Leaves on the lower half of each cutting were removed to reduce transpiration and support rooting.

This preparation ensured consistent physiological status and environmental conditions, facilitating a reliable evaluation of the effects of auxin type and concentration on root initiation.

### 2.2. Hormone Treatments

Three different auxins from the indole and naphthalene families were used in this study: IBA, NAA, and IAA (all obtained from Merck, Sigma-Aldrich, Darmstadt, Germany). Each auxin was tested at four concentrations: 1000, 3000, 5000, and 8000 ppm. The hormones were initially dissolved in 96% ethanol (Merck, Sigma-Aldrich, Darmstadt, Germany) and then thoroughly mixed with pharmaceutical-grade talcum powder (Merck, Sigma-Aldrich, Darmstadt, Germany) to obtain uniform powdered formulations.

Prior to planting, the base end (approximately 1–2 cm) of each cutting was washed with pure water and dipped in the relevant auxin powder for 3–4 s to ensure consistent hormonal contact. A control group (no hormone treatment) was also included for comparison. This powder-based application method was chosen for its practicality and extended auxin availability at the rooting site, which has been reported to enhance root initiation efficiency in semi-hardwood ornamental cuttings.

### 2.3. Rooting Medium and Experimental Setup

The treated cuttings were vertically inserted into plastic trays filled with sterile perlite (Metaper, Istanbul, Türkiye), which served as the rooting medium due to its excellent aeration and moisture retention properties. The trays were placed on bottom-heated propagation benches maintained at a constant temperature of 25 ± 2 °C to stimulate root development. The bottom-heated propagation benches were set to maintain a root-zone temperature of approximately 25 ± 2 °C; however, actual substrate (root zone) temperatures were monitored and recorded independently throughout the experiment. Root-zone temperatures were manually measured within the perlite medium at a depth of 5 cm using calibrated thermometers (Hanna Instruments HI98331 soil conductivity meter, Temse, Belgium) throughout the 120-day rooting period, with a total of 30 measurements taken at 4-day intervals. These temperatures ranged between 18 °C and 22 °C, with an average of 20 ± 2 °C. For each hormone group, the mean temperature of the 30 recordings was calculated and used in the statistical analyses and regression models. Relative humidity was maintained at 70 ± 2% throughout the rooting period to ensure consistent greenhouse moisture conditions. Intermittent misting was applied to prevent desiccation and maintain consistent moisture levels throughout the rooting period. No foliar nutrients or microbial agents were applied during the rooting period.

The experimental layout followed a “randomized complete block design (RCBD)” with 3 replicates per treatment, each consisting of 20 cuttings. The basal ends of the cuttings were first moistened with distilled water. Immediately afterward, only the freshly cut surface (1–2 mm) of each cutting was gently touched to the auxin powder for approximately 3 s to ensure uniform application. The design included three auxin types (IBA, NAA, and IAA), four concentrations (1000, 3000, 5000, and 8000 ppm), and a control group, resulting in a total of 780 cuttings (3 hormones × 4 doses × 3 replicates × 20 cuttings + control). The selected auxin concentrations were based on prior literature and informal preliminary trials, aiming to cover a wide range from suboptimal to potentially inhibitory levels. No additional fertilizers or microbial additives were applied during the rooting period to ensure that all observed effects were attributable solely to the auxin treatments.

### 2.4. Data Collection

After approximately 120 days in the propagation environment, the following parameters were measured for each cutting:

- Rooting percentage (%): Calculated as the number of rooted cuttings divided by the total number of cuttings per treatment, multiplied by 100.
- Average number of roots per rooted cutting: All primary roots were counted.
- Average root length (cm): The longest root of each cutting was measured using a precision ruler with 1 mm accuracy.
- Callus formation rate (%): Visual observation of undifferentiated tissue development at the basal end of the cuttings.
- Mortality rate (%): The proportion of cuttings that showed no root or callus formation and were classified as decayed or desiccated.

All measurements were taken at the end of the propagation period, and standardized data sheets were used to ensure consistency across replicates.

### 2.5. Statistical Analysis

The recorded data were initially analyzed using one-way analysis of variance (ANOVA) in SPSS 23.0 (IBM Corp., Armonk, NY, USA) to assess the significance of individual treatments. Mean comparisons were performed using Tukey’s honest significant difference (HSD) test at the 5% significance level (*p* < 0.05), and results were presented as mean ± standard deviation (SD). Graphical outputs were generated using Microsoft Excel (Microsoft Corp., Redmond, WA, USA).

To further explore the interactions among independent variables (temperature, auxin type, and concentration), response surface methodology (RSM) was employed using central composite design (CCD). In this study, response surface methodology (RSM) was preferred over classical analysis of variance. Unlike ANOVA, RSM enables not only the identification of factor effects and their interactions, but also the modeling and visualization of the response surface, allowing for prediction of optimal treatment combinations. Therefore, it provides more comprehensive insights and practical applicability compared to a standard multi-way factorial analysis. Regression models were developed for rooting percentage (R), callus formation (K), and root length (RM) using second-order polynomial equations. Model adequacy was validated via ANOVA, residual diagnostics, and coefficient of determination (R^2^). Diagnostic plots, including Pareto charts, contour plots, and normal probability plots, were used to verify statistical assumptions, such as normality, homoscedasticity, and independence.

### 2.6. Data Recording, Preliminary Analysis, and Model Design

The data obtained from the experimental applications conducted in this study were systematically recorded and organized for statistical analysis. In order to minimize experimental errors and ensure data consistency, all measurement results were entered into standardized data collection forms and digital Excel spreadsheets. Environmental parameters, such as humidity and light intensity, were maintained at constant levels during the rooting period to improve data reliability.

In the preliminary analysis phase, descriptive statistics were applied to assess the validity of the observed values, and any outliers were identified and handled using appropriate methods to reduce systematic or recording errors. Measurement reliability was enhanced through repeated measurements at regular intervals for each parameter.

In the second stage, response surface methodology (RSM) was employed to explore and optimize the interactions between multiple variables. Specifically, a central composite design (CCD) was adopted to model the effects of hormone type (H), concentration (ppm), and substrate (root zone) temperature (T) on the dependent variables—rooting percentage (R), callus formation rate (K), and root length (RM) [38].

The general form of the mathematical regression model used in this study is expressed as follows:

(1) $$Y=a_{0}+\sum_{i=1}^{k}b_{i}X_{i}+\sum_{ij}^{k}b_{ij}X_{ij}+\sum_{i=1}^{k}b_{ii}X_{i}^{2}+\cdots \;n$$

These models were developed to statistically predict the plant development parameters under different hormonal and environmental conditions. Model outputs were validated by comparing predicted values with observed experimental data. This validation process aimed to test model reliability and improve its predictive accuracy under variable parameter combinations.

## 3. Results

### 3.1. Effect of Auxin Type and Concentration on Rooting Percentage

**Figure 1:**
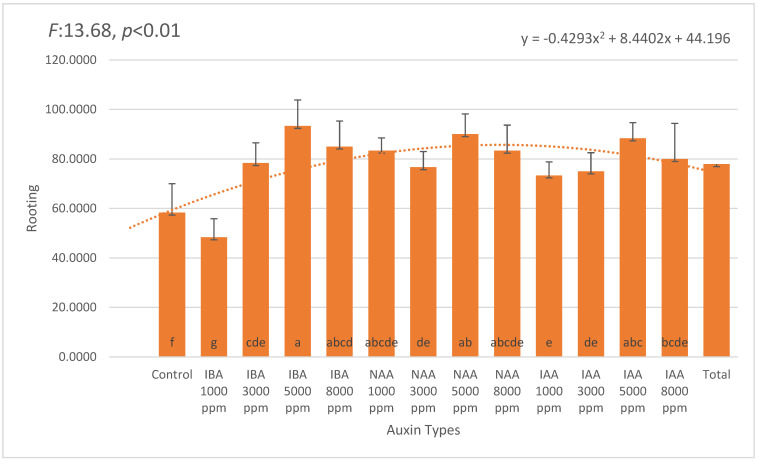
Rooting percentages (mean ± SD) of *Photinia × fraseri* cuttings treated with different auxin types and concentrations (1000–8000 ppm). The highest rooting rate was observed with 5000 ppm IBA (93.33 ± 8.16%), followed by 5000 ppm NAA (90.00 ± 6.32%) and 5000 ppm IAA (88.33 ± 7.53%). The lowest rooting rate was recorded in the control group (58.33 ± 7.53%) and 1000 ppm IBA treatment (48.33 ± 11.69%), indicating that auxin application significantly improved rooting performance. Error bars represent standard deviation. A quadratic trendline was fitted to illustrate the overall response pattern across treatments. Different letters above the bars indicate statistically significant differences (*p* < 0.05). The orange dotted line represents the trend line of the second-degree (quadratic) equation.

The rooting performance of *Photinia × fraseri* stem cuttings showed a statistically significant variation based on auxin type and concentration (*p* < 0.05). Among the three auxins tested—IBA, NAA, and IAA—IBA consistently resulted in the highest rooting percentages across all applied concentrations. The most effective treatment was 5000 ppm IBA, which achieved an average rooting rate of 93.33%, a statistically significant increase compared to the control (58.33%; Figure 1).

In contrast, NAA treatments produced comparable rooting success, with 5000 ppm NAA reaching 90.00%, followed by 1000 ppm and 8000 ppm NAA (both 83.33%). The lowest NAA value was at 3000 ppm (76.67%), suggesting a relatively broad concentration window for effective rooting. IAA treatments also yielded favorable results, with 5000 ppm IAA achieving 88.33%, followed by 1000 ppm and 8000 ppm (both 80.00%), and 3000 ppm with 75.00%. Although IAA is often considered less stable in exogenous applications, its performance in this study remained relatively consistent, with all tested concentrations producing over 75% rooting.

### 3.2. Effect on Root Number and Root Length

**Figure 2:**
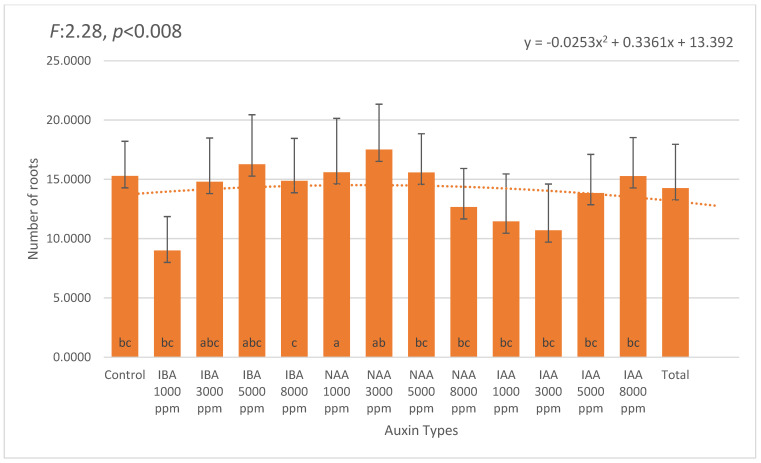
Mean number of roots per *Photinia* × *fraseri* cutting under different auxin types and concentrations (1000–8000 ppm). Error bars indicate standard deviation. The highest average root number was observed in the 3000 ppm NAA treatment (6.17), followed by 3000 ppm IBA (5.96) and 5000 ppm IBA (5.75). In contrast, the 8000 ppm concentrations of NAA (4.50) and IAA (4.88), as well as the control group (5.03), exhibited lower root initiation. A second-order polynomial trendline illustrates the overall trend across treatments. Different letters above the bars indicate statistically significant differences (*p* < 0.05). The orange dotted line represents the trend line of the second-degree (quadratic) equation.

The number of roots per cutting followed a pattern generally consistent with rooting percentage. The highest average root number was observed in 3000 ppm NAA (6.17), followed by 3000 ppm IBA (5.96) and 5000 ppm IBA (5.75). The control group averaged 5.03 roots, indicating that while exogenous auxin stimulation enhanced rooting performance, basal root formation was also possible without treatment (Figure 2).

**Figure 3:**
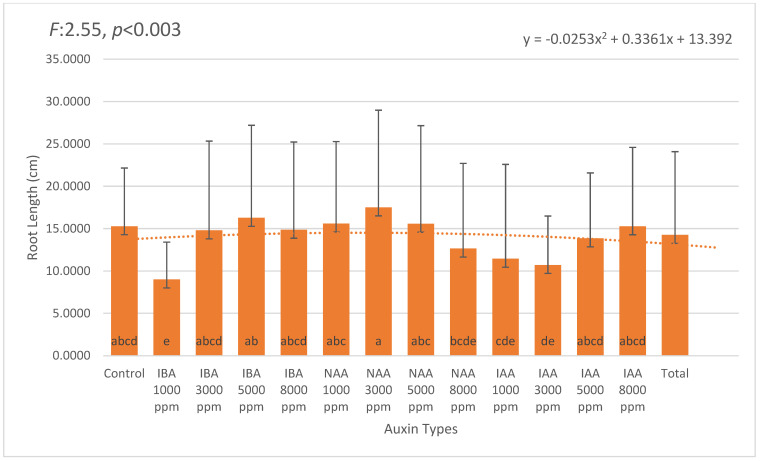
Mean root length (cm) in *Photinia × fraseri* cuttings under different auxin treatments. Error bars represent standard deviation. The longest roots were observed in the 3000 ppm NAA treatment (17.51 cm), followed by 5000 ppm IBA (16.27 cm) and 8000 ppm IAA (15.28 cm). In contrast, shorter roots were recorded at lower concentrations, such as 1000 ppm IBA (9.00 cm) and 3000 ppm IAA (10.71 cm), indicating that auxin concentration plays a critical role in root elongation. A second-order polynomial trendline illustrates the overall variation pattern across treatments. Different letters above the bars indicate statistically significant differences (*p* < 0.05). The orange dotted line represents the trend line of the second-degree (quadratic) equation.

Average root length was also significantly affected by the treatments. The longest roots were recorded in 3000 ppm NAA (17.51 cm), followed by 5000 ppm IBA (16.28 cm) and 1000 ppm NAA (15.60 cm). In contrast, the shortest roots were observed in 1000 ppm IBA (9.00 cm) and 3000 ppm IAA (10.71 cm), suggesting possible auxin-specific responses in elongation dynamics (Figure 3).

### 3.3. Callus Formation and Mortality Rates

**Figure 4:**
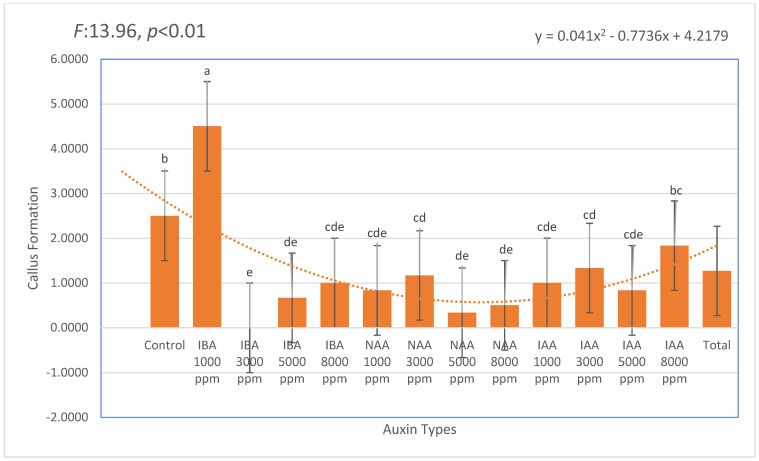
Effect of different auxin types and concentrations on callus formation in *Photinia × fraseri* stem cuttings. Bars indicate mean ± standard deviation. A second-order polynomial trendline illustrates the overall response pattern. Significant differences were observed among treatments. Different lowercase letters placed above the error bars indicate statistically significant differences among treatments (*p* < 0.05). The orange dotted line represents the trend line of the second-degree (quadratic) equation.

Callus formation was observed across all auxin treatments, and the data were calculated based on the number of cuttings that did not root but exhibited visible callus development out of 60 stem cuttings per group. The most intense callusing was recorded in the IBA 1000 ppm treatment (mean: 4.50), followed by the untreated control (mean: 2.50). Notably, IAA treatments—particularly at 8000 ppm (mean: 1.83) and 3000 ppm (mean: 1.33)—showed moderate callus formation, though this did not consistently translate into successful rhizogenesis. These findings suggest that while certain auxin concentrations stimulate cellular activity leading to callus production, this does not necessarily result in organized root development. Mortality remained low across most treatments, with the lowest rates (6.67–13.33%) recorded in the 3000–5000 ppm IBA range. These results indicate that IBA not only enhanced rooting efficiency but also minimized physiological stress, supporting its role as a safe and effective auxin for the vegetative propagation of *Photinia* × *fraseri* (Figure 4).

### 3.4. Overall Performance Ranking of Treatments

When considering rooting percentage, root number, and root length collectively, the treatment of 5000 ppm IBA demonstrated the most consistent and superior performance for the vegetative propagation of *Photinia × fraseri*. While 3000 ppm NAA showed the longest average root length (17.51 cm), it was less effective in other parameters. IAA, particularly at higher concentrations, was associated with variable results and reduced rooting efficiency, likely due to degradation and instability under exogenous conditions.

### 3.5. RSM-Based Optimization and Model Validation

To complement the classical one-way ANOVA results, a response surface methodology (RSM) approach was applied to model and optimize the effects of substrate (root-zone) temperature (T), auxin concentration (ppm), and hormone type (H) on three response variables: rooting percentage (R), callus formation rate (K), and root length (RM). The central composite design (CCD) method was used for experimental design, and second-order polynomial regression models were developed and validated using experimental data.

#### 3.5.1. Rooting Percentage (R)

The ANOVA results (Table 1) revealed that the constructed model explained 92.79% of the total variance in rooting percentage, with a model F-value of 93.01, indicating strong model performance. The most influential linear terms were temperature (27.65%) and auxin concentration (25.88%). Square and interaction terms also contributed meaningfully, particularly the ppm^2^ term. The model was validated using regression equations and residual analysis, confirming its reliability.

The ANOVA results presented in Table 1 indicate that the constructed model successfully explained 92.79% of the total variance, demonstrating a high level of explanatory power. The model’s F-value of 93.01 suggests a strong effect on the dependent variable. The linear terms alone accounted for 68.82% of the variance, with temperature (T) and auxin concentration (ppm) emerging as the most influential factors, contributing 27.65% and 25.88%, respectively. This highlights the substantial impact of these two parameters on system behavior. Quadratic terms and two-way interactions contributed 12.33% and 6.49%, respectively, with the squared concentration term (ppm^2^) showing a relatively high effect (F = 11.15). The low error rate (17.21%) and the low adjusted mean square error (Adj. MS = 29.314) further support the overall reliability of the model. These results indicate that the regression model is in strong agreement with the experimental data and serves as a dependable tool for predicting the dependent variable.

**Figure 5:**
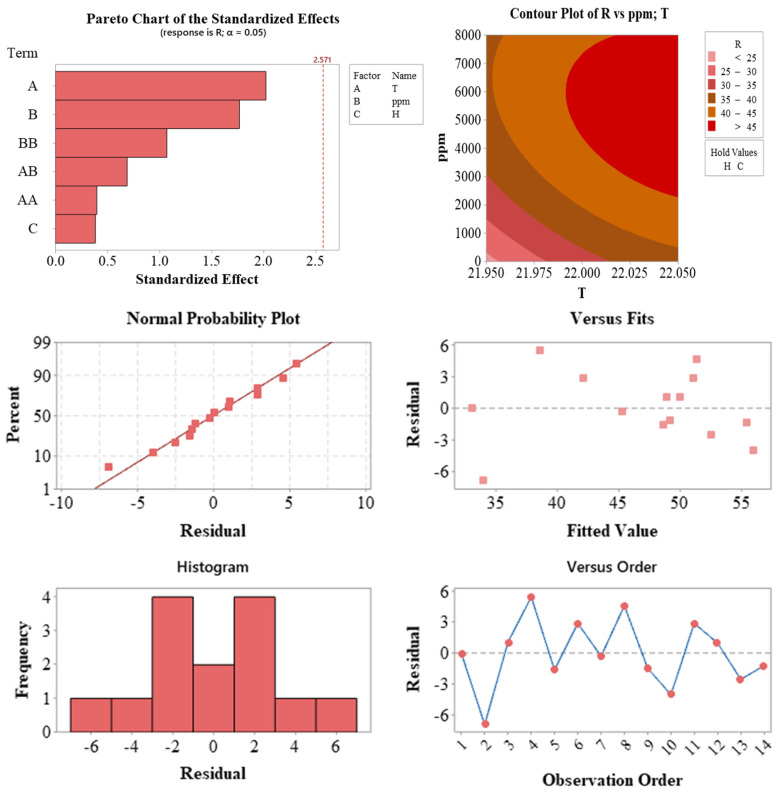
Statistical validation outputs of the regression model used for optimizing the propagation of *Photinia × fraseri* cuttings. The Pareto chart (**top left**) displays the standardized effects of model terms. The contour plot (**top right**) illustrates the interaction between temperature (T) and auxin concentration (ppm) on the response variable. The normal probability plot of residuals (**middle left**), residuals vs. predicted values (**middle right**), residuals vs. observation order (**bottom right**)**,** and residual histogram (**bottom left**) collectively verify the model’s assumptions of normality, independence, and homoscedasticity.

Figure 5 illustrates the statistical validation outputs of the regression model and the effects of influencing factors, such as temperature and auxin concentration, on the response variable.

The statistical analysis results presented in Figure 5 illustrate the validity of the model and the effects of influencing factors. The Pareto chart (top left) displays the standardized effect sizes of each factor. Although no term exceeded the 5% significance threshold (indicated by the red line), temperature (A) and auxin concentration (ppm, B) exhibited effect sizes that approached this threshold, supporting the high contribution rates observed in the ANOVA.

The contour plot (top right) visualizes the combined effects of temperature (T) and auxin concentration (ppm) on the response variable (R). This plot reveals that higher response values can be achieved in regions with low temperature and high auxin concentration, providing valuable guidance for optimizing propagation parameters.

Several residual plots were used to assess the quality of the model. The normal probability plot (middle left) indicates that the residuals followed a largely linear pattern, suggesting that the assumption of normality was met. The plots of residuals versus predicted values (middle right) and observation order (bottom right) showed no systematic trends or increases in variance, confirming the assumptions of homoscedasticity and independence. The residual histogram (bottom left) displayed a symmetrical and centrally clustered distribution.

Collectively, these plots demonstrate that the model is statistically reliable and consistent with fundamental assumptions. Furthermore, they highlight the significant influence of production parameters, such as temperature and auxin concentration, on the response variable.

#### 3.5.2. Callus Formation (K)

According to Table 2, the model explained 89.57% of the variance in callus formation. Temperature and hormone type were identified as key contributors. While the model’s overall F-value (2.43) and *p*-value (0.171) did not reach high statistical significance, the model still provided practical predictive value with acceptable error margins (20.43%).

The analysis of variance (ANOVA) results presented in Table 2 indicate that the constructed model had a considerable level of explanatory power for the dependent variable. The model accounted for 89.57% of the total variance, demonstrating a strong overall fit. The linear terms contributed a total of 60.67%, with temperature (T) and hormone type (H) contributing 20.13% and 36.75%, respectively, indicating their significant influence on the dependent variable. The quadratic terms explained 17.24% of the variance, with the squared temperature term (T^2^) contributing 14.23%, which is particularly noteworthy. Two-way interactions were less influential, with the T×ppm interaction accounting for 1.66% of the variance.

The model’s F-value was 2.43, with a corresponding *p*-value of 0.171, which, while slightly above the conventional threshold for statistical significance, still indicates an acceptable level of reliability. The error rate was 20.43%, and the adjusted mean square error (Adj. MS = 10.4382) remained low, supporting the model’s predictive performance. These findings suggest that environmental parameters, such as temperature and hormone type, have meaningful effects on the dependent variable and that the model represents the experimental data to a satisfactory degree. Moreover, the low error value further confirms the reliability and accuracy of the statistical analysis.

**Figure 6:**
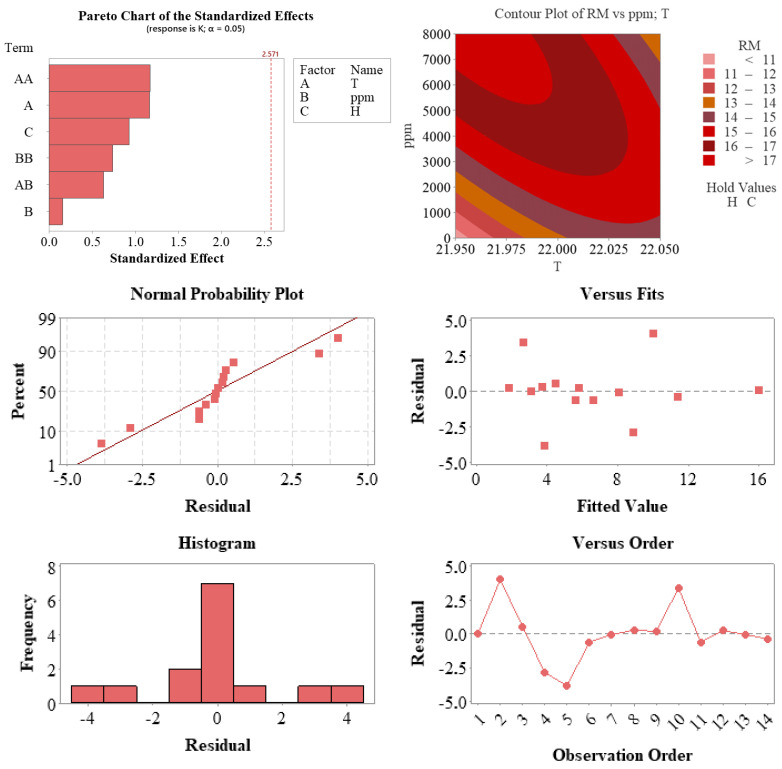
Graphical diagnostics and validation of the regression model developed for root length (RM). The Pareto chart (**top left**) displays standardized effects of the model terms, highlighting temperature (A) and squared temperature (AA) as the most influential. The contour plot (**top right**) illustrates the interaction between root-zone temperature and auxin concentration on RM. Residual analysis plots—including the normal probability plot (**middle left**), residuals vs. predicted values (**middle right**), observation order plot (**bottom right**), and residual histogram (**bottom left**)—demonstrate compliance with assumptions of normality, independence, and homoscedasticity, supporting the statistical validity and predictive strength of the model.

Figure 6 presents the graphical outputs used to validate the second regression model and to evaluate the effects of temperature and auxin concentration on root length (RM).

The statistical evaluations presented in Figure 6 were developed to assess the validity of the model and analyze the effects of the factors on the response variable (RM). The Pareto chart shows that while no term exceeded the 5% significance threshold, the temperature (A) and squared temperature (AA) terms stood out with standardized effect values close to the significance limit. This indicates that temperature, in particular, had a notable impact on the response variable.

The contour plot for the T and ppm variables revealed an upward trend in the response values within the interaction zones of these two parameters. The graph demonstrates that root length (RM) approached its maximum under moderate temperature and high auxin concentration, providing valuable guidance for process optimization.

Regarding the residual analysis of the model, the normal probability plot indicates that the residuals largely followed a straight line, confirming the assumption of normality. The residuals versus fitted values and observation order plots showed no systematic bias or trend, suggesting that the assumptions of homoscedasticity and independence were satisfied. The histogram also showed a symmetric, centrally clustered distribution of residuals.

Collectively, these findings confirm that the model was consistent with statistical assumptions and reliably represented the experimental data. Temperature and auxin concentration appeared to be key determinants of system behavior and directly contributed to the model’s performance.

#### 3.5.3. Root Length (RM)

As presented in Table 3, the model accounted for 80.37% of the variance in root length, with particularly high contributions from temperature^2^ (31.66%) and the T×ppm interaction term (23.83%). Although the F-value was moderate (2.56), and the *p*-value was slightly above the threshold (0.158), the model demonstrated useful predictive performance.

The analysis of variance (ANOVA) results presented in Table 3 indicate that the model possessed a generally high level of explanatory power. The model accounted for 80.37% of the total variance, demonstrating a strong predictive capacity. The linear terms contributed a total of 23.92%, with temperature (T) and hormone type (H) standing out, contributing 11.07% and 10.21%, respectively. The auxin concentration (ppm) had a limited effect, contributing only 2.64%.

The quadratic terms explained 32.62% of the variance, with the squared temperature term (T^2^) alone contributing 31.66%, which is particularly noteworthy. Two-way interactions also played a significant role in the model, with the T×ppm interaction accounting for 23.83% of the total variance. This interaction yielded an F-value of 6.07 and a *p*-value of 0.057, which is very close to the threshold of statistical significance, suggesting that the interaction between temperature and auxin concentration may have a considerable impact on the dependent variable.

The model’s F-value of 2.56 and *p*-value of 0.158 indicate that the model was close to the level of statistical significance. The error rate of 19.63% and the relatively low adjusted mean square error (Adj. MS = 2.4997) further supported the model’s reliability.

In conclusion, the established model adequately represented the experimental data and highlighted the critical role of quadratic terms and interactions in understanding system behavior. These findings suggest that considering both temperature and auxin concentration together can significantly enhance process optimization efforts.

**Figure 7:**
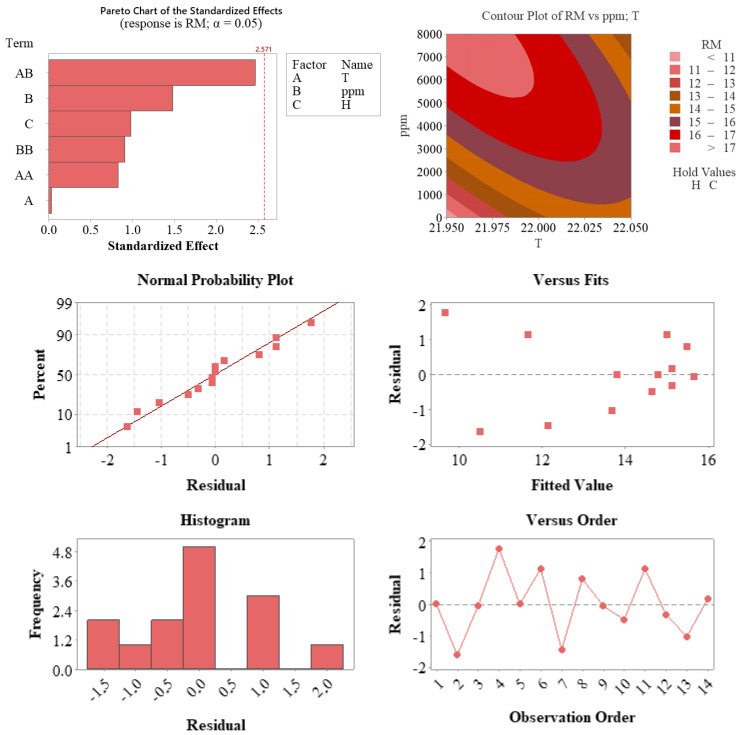
Diagnostic and interaction plots supporting the regression model developed for root length (RM). The Pareto chart (**top left**) highlights the interaction between temperature and auxin concentration (AB) as the most influential factor, with additional contributions from auxin concentration (ppm) and hormone type (H). The contour plot (**top right**) visualizes the combined effect of temperature and auxin concentration on RM, showing an optimal zone at moderate temperature and high auxin levels. The residual diagnostics—including the normal probability plot (**middle left**), residuals vs. predicted values (**middle right**), residual histogram (**bottom left**), and residuals vs. observation order (**bottom right**)—confirm that the model satisfies statistical assumptions of normality, homoscedasticity, and independence.

Figure 7 provides a comprehensive graphical representation of the model diagnostics and interaction effects between temperature and auxin concentration on root length (RM).

According to the statistical analysis graphs presented in Figure 7, the validity of the model and the effects of the parameters on the response variable (RM) were comprehensively evaluated. The Pareto chart (top left) shows that the interaction between temperature and auxin concentration (AB) was the closest term to the significance threshold (2.571), indicating that this interaction was the most influential factor in the model. Additionally, auxin concentration (ppm) and hormone type (H) also contributed meaningfully in terms of standardized effect sizes.

The contour plot (top right) illustrates the two-dimensional effect of the interaction between temperature (T) and auxin concentration (ppm) on RM. The graph reveals that the response variable increased notably under conditions of high auxin concentration and moderate temperature. This supports the importance of jointly evaluating these two parameters in process optimization.

Regarding residual analysis for model quality, the normal probability plot (middle left) shows that the residuals followed a largely linear pattern, confirming the assumption of normal distribution. The residuals versus predicted values plot (middle right) demonstrates a random distribution without systematic bias, and the histogram (bottom left) shows a symmetrical and centrally concentrated distribution of residuals. The residuals versus observation order plot (bottom right) confirms the absence of time-related error trends.

These findings indicate that the developed model was consistent with statistical assumptions and accurately represented the experimental data. In particular, the interaction between auxin concentration and temperature had a significant effect on the response variable, providing valuable guidance for model-based process optimization.

#### 3.5.4. Diagnostic Plots and Model Assumptions

Diagnostic tools, including Pareto charts, contour plots, and residual analyses (Figure 5, Figure 6 and Figure 7), confirmed that the models adhered to statistical assumptions, such as normality, homoscedasticity, and independence. Notably, the T×ppm interaction emerged as a significant factor influencing RM, highlighting the importance of evaluating parameter interactions in propagation studies.

These findings reinforce the utility of RSM as a robust tool for modeling complex biological processes, enabling systematic optimization of propagation parameters. The models established in this study provide a reliable framework for predicting and improving rooting performance in *Photinia × fraseri* under varying hormonal and environmental conditions.

## 4. Discussion

The findings of this study confirm that the rooting efficiency of *Photinia × fraseri* stem cuttings was significantly influenced by both the type and concentration of applied exogenous auxins. Among the three tested auxins—IBA, NAA, and IAA—IBA emerged as the most effective in promoting adventitious root formation, as evidenced by superior performance across all measured parameters: rooting percentage, root number, and root length. The application of 5000 ppm IBA resulted in the highest rooting percentage (93.33%) and produced robust root systems in terms of both root number (5.75) and length (16.27 cm). Although the maximum average root number was observed at 1000 ppm NAA (7.06), this difference was not statistically significant. Furthermore, the longest average root length was recorded with 3000 ppm NAA (17.51 cm), suggesting a concentration-specific enhancement in root elongation. These findings are consistent with a broad range of studies recognizing IBA as the most reliable auxin for promoting adventitious rooting, particularly in semi-hardwood ornamental cuttings, such as *Hedera algeriensis* [39], *Camellia japonica* [6], *Linum perenne* [12], and *Linum* spp. [16]. The stability of IBA, along with its lower sensitivity to oxidative degradation, likely contributes to its effectiveness under propagation conditions [15,40]. Consistent results have also been reported in *Cistus creticus* and *Bougainvillea glabra*, where IBA treatments significantly enhanced rooting success and plant survivability under controlled propagation conditions [33,34,41]. However, the data also indicated that excessively high concentrations (8000 ppm) of IBA led to reduced rooting success and increased callusing, which is consistent with auxin over-accumulation effects seen in *Magnolia* spp. and *Rosa hybrida* [42,43]. This biphasic response aligns with the classic auxin dose–response curve, wherein optimal concentration windows exist for maximum rooting efficacy [14,18]. Similarly, high concentrations of NAA and IAA (8000 ppm) resulted in stunted root elongation or malformed root structures, reflecting potential phytotoxic effects reported at supra-optimal auxin doses [14,18,42]. NAA demonstrated high rooting efficiency at 3000 ppm (76.66%), though this declined with increasing concentrations. This trend is echoed in the work of Kaushik and Shukla [42] and Tan [31], who reported that NAA induced variable rooting and was more prone to stimulate excessive callus tissue rather than structured root primordia. The tendency of NAA to favor callogenesis over rhizogenesis suggests a hormonal pathway divergence, which has also been noted in leguminous species under stress [32,36]. IAA demonstrated the least consistent rooting response, with a relatively narrow effective range and only moderate success at 3000 ppm (75.00%). Although a high rooting rate (80.00%) was recorded at 8000 ppm, this treatment exhibited a high degree of callus formation and inconsistent root development, limiting its practical efficacy. This pattern correlates with the known instability of IAA in exogenous conditions, due to its rapid photooxidative and enzymatic degradation [19,40]. Similar shortcomings of IAA were reported in the propagation of foliage plants, such as *Chrysanthemum morifolium* and *Apocynum lancifolium* [44,45]. Beyond hormonal influence, environmental conditions, such as misting regime and shading, likely contributed to the overall success. These factors are recognized as critical for maintaining appropriate turgor and oxygenation, which are essential for root development [18,20,22,46,47]. Studies by Davis and Potter [3] and Lee et al. [16] support this view, emphasizing the synergistic role of environmental microclimate and auxin signaling. It is important to note that rooting responses were observed under stable environmental conditions, in which root-zone temperatures—measured every 4 days throughout the 120-day experimental period—were consistently maintained within a narrow range of 18–22 °C. These controlled conditions minimized external variability and allowed clearer evaluation of auxin-specific effects on rooting parameters. This finding aligns with broader urban-scale studies that emphasize the critical role of vegetation and green infrastructure in regulating microclimatic parameters, such as temperature and humidity—factors that also directly influence plant physiological responses and propagation efficiency [30]. The high callus formation observed in NAA and IAA treatments—particularly at 5000–8000 ppm—without corresponding root development underscores that callogenesis does not guarantee rhizogenesis. Similar patterns were reported in *Bougainvillea* tissue culture trials [33], and in vegetative propagation of yam, where callus often remained unorganized or regressed [21,48]. The high callus formation observed in IAA treatments, especially at elevated concentrations, without corresponding root development, may reflect hormonal imbalance or inadequate signaling for rhizogenesis, and suggests a potential optimization target for future protocols [25]. The untreated control consistently exhibited the weakest performance across all parameters. These outcomes strongly emphasize the importance of auxin selection and concentration optimization in improving propagation efficiency for this species. Furthermore, the results reinforce literature suggesting the species-specific responsiveness of ornamental shrubs to hormonal manipulation [39,42,49,50].

While these results are promising under controlled conditions, future research is necessary to assess their reproducibility under variable environmental settings and across other woody ornamental species. Such comparative studies would help generalize the propagation protocols and refine them for broader commercial application. The identification of 5000 ppm IBA as the optimal treatment presents a promising strategy for improving rooting success in nursery production. However, scaling this concentration may involve considerations related to material cost and operational logistics. To enhance field applicability, future efforts may explore cost-effective formulations, simplified application protocols (e.g., quick-dip methods), or synergy with natural biostimulants that reduce dependence on higher synthetic auxin doses. From a commercial and ecological perspective, the identification of 5000 ppm IBA as the optimal treatment for *Photinia × fraseri* Dress. cuttings presents a cost-effective and efficient propagation protocol [6,51,52,53]. It also addresses a significant knowledge gap, as most prior research on *Photinia* sp. propagation focused on general horticultural outcomes without comparative hormonal analysis [5,7,9,54]. The species-specific rooting response profile outlined here not only informs nursery practices but also opens pathways for advanced rooting models using integrated biostimulant–hormone systems, as proposed in recent PGPR studies [25,35,55].

### Comparative Evaluation of the Three RSM Models

When the ANOVA results and statistical validation tests of the three different RSM models were evaluated together, it was evident that all models exhibited a high level of explanatory power for their respective dependent variables. The model in Table 1 showed the highest level of fit, explaining 92.79% of the total variance, followed by the models in Table 2 (89.57%) and Table 3 (80.37%). This suggests that while all models reliably represented the experimental data, the first model had the strongest predictive capacity. In terms of linear terms, temperature (T) and auxin concentration (ppm) emerged as dominant factors in all models, contributing more than 53% in total in Table 1 alone. Regarding quadratic terms, the squared temperature (T^2^) component in the second and third models showed substantial contributions of 14.23% and 31.66%, respectively. This indicates nonlinear system behavior and highlights the importance of second-order effects in improving model performance. Among the interaction terms, the most notable result was observed in Table 3, where the T×ppm interaction contributed 23.83% to the model and had a *p*-value of 0.057, indicating a near-significant effect. This underscores the critical role of the temperature and concentration interaction in understanding system dynamics.

Residual analysis results for all models demonstrated similar patterns. Normal probability plots indicated that residuals were generally normally distributed, histograms showed symmetrical and centrally concentrated distributions, and no systematic deviations were observed in the residuals versus fitted values or observation order plots. These results confirm that the assumptions of independence and homoscedasticity were met. In conclusion, all three models were statistically reliable, compatible with the experimental data, and exhibited high predictive power. While the model in Table 1 stood out in terms of overall fit, the model in Table 3 offered meaningful contributions through second-order and interaction terms. These findings demonstrate that different modeling approaches reveal various aspects of system behavior and evaluating them together provides a more comprehensive analysis. The combined consideration of temperature, auxin concentration, and their interaction emerged as a key strategy for system optimization. Through the RSM analysis, not only the individual effects of the factors but also the interactions between auxin type and concentration were evaluated. By modeling these interactions, the influence of specific auxin–dose combinations on the rooting success of cuttings was more clearly revealed, allowing the identification of optimal combinations. This approach goes beyond merely detecting statistically significant differences and contributes to decision-making processes aimed at practical applications. The regression equations derived for all three models describe the relationship between the dependent variable and the independent variables temperature (T), auxin concentration (ppm), their squared terms, and their interaction. The general form of the regression equation is as follows:

Y = β_0_ + β_1_T + β_2_ppm + β_3_T^2^ + β_4_ppm^2^ + β_5_T⋅ppm,

where:

Y = response variable (R, K, or RM),

Β_0_ = intercept,

Β_1_, β_2_, etc. = regression coefficients.

T = temperature,

ppm = auxin concentration.

This equation structure reflects not only linear relationships but also nonlinear and interaction-based behaviors in the system. For example, the regression equation for Table 1 is expressed as:

R= − 419732 + 38014T + 0.227⋅ppm − 861T^2^ − 0.000000⋅ppm^2^ − 0.0101T⋅ppm.

Here, positive coefficients (e.g., 38,014T) indicate enhancing effects on the response variable, while negative coefficients (e.g., −861T^2^) reflect diminishing marginal returns beyond certain thresholds. This supports the idea that parameters such as temperature may have optimal values and exceeding those can negatively impact the system. Additionally, the interaction term (T·ppm) highlights that the combined effect of these variables may differ from their individual effects. The similarity in model structure across all equations allows for direct parametric comparisons, offering significant advantages in process optimization. While temperature and auxin concentration appear as primary influencing factors in all models, the inclusion of quadratic and interaction terms enhances model explanatory power. This framework enables statistically sound modeling of the propagation system and provides a basis for reliable predictions under varying conditions.

## 5. Conclusions

This study investigated the effects of three auxin types (IBA, NAA, and IAA) at four concentrations (1000, 3000, 5000, and 8000 ppm) on the rooting performance of *Photinia × fraseri* stem cuttings under controlled greenhouse conditions (where root-zone temperatures were consistently maintained between 18 and 22 °C and relative humidity was held at 70 ± 2% throughout the 120-day rooting period). Among the treatments, 5000 ppm IBA produced the highest rooting percentage (93.33%) and exhibited strong performance in both root number and root length, confirming its superior efficacy as an exogenous auxin in woody plant propagation. Although the maximum root number and length were observed with 1000 ppm and 3000 ppm NAA, respectively, these differences were not always statistically significant compared to 5000 ppm IBA. In contrast, higher concentrations of NAA and IAA negatively affected rooting, likely due to phytotoxic effects or hormonal instability. The sterile perlite rooting medium contributed positively to root development by ensuring proper aeration and moisture retention. While gene expression was not directly analyzed, the differential effectiveness of each auxin type reflects their known regulatory roles in activating root primordium formation and auxin-responsive gene pathways. If integrated into future protocols, response surface methodology (RSM) could enable predictive modeling of hormone–response interactions, helping to optimize vegetative propagation strategies with minimal trial-and-error. Future studies may benefit from incorporating molecular tools to explore auxin-mediated gene expression or evaluating alternative substrate–environment combinations to refine propagation strategies for *Photinia × fraseri* and other ornamental shrubs.

## Figures and Tables

**Table 1:** Analysis of variance.

Source	DF	Seq. SS	Contribution	Adj. SS	Adj. MS	F-Value	*p*-Value
Model	8	705.15	92.79%	705.146	88.143	93.01	0.120
Linear	5	586.15	68.82%	659.102	131.820	44.50	0.062
T	1	235.52	27.65%	119.722	119.722	24.08	0.099
ppm	1	220.43	25.88%	91.469	91.469	33.12	0.138
H	3	130.20	15.29%	41.354	13.785	20.47	0.416
Square	2	105.05	12.33%	83.445	41.722	1.42	0.324
T×T	1	77.63	9.11%	4.660	4.660	0.16	0.507
ppm×ppm	1	27.42	3.22%	33.619	33.619	11.15	0.333
2-Way Interaction	1	13.94	1.64%	13.944	13.944	0.48	0.421
T×ppm	1	13.94	1.64%	13.944	13.944	0.48	0.421
Error	5	146.57	17.21%	146.568	29.314		
Total	13	851.71	100.00%				
Regression Equation in Uncoded Units							
H							
C	R	=	−419,732 + 38,014 T + 0.227 ppm − 861 T×T − 0.000000 ppm×ppm − 0.0101 T×ppm				
IAA	R	=	−419,722 + 38,014 T + 0.227 ppm − 861 T×T − 0.000000 ppm×ppm − 0.0101 T×ppm				
IBA	R	=	−419,726 + 38,014 T + 0.227 ppm − 861 T×T − 0.000000 ppm×ppm − 0.0101 T×ppm				
NAA	R	=	−419,725 + 38,014 T + 0.227 ppm − 861 T×T − 0.000000 ppm×ppm − 0.0101 T×ppm				

**Table 2:** Analysis of variance.

Source	DF	Seq. SS	Contribution	Adj. SS	Adj. MS	F-Value	*p*-Value
Model	8	203.237	89.57%	203.237	25.4047	2.43	0.171
Linear	5	154.976	60.67%	146.665	29.3330	2.81	0.141
T	1	51.429	20.13%	14.203	14.2032	1.36	0.296
ppm	1	9.669	3.79%	0.261	0.2615	0.03	0.480
H	3	93.878	36.75%	38.130	12.7100	1.22	0.394
Square	2	44.032	17.24%	48.262	24.1308	2.31	0.195
T×T	1	36.342	14.23%	14.342	14.3416	1.37	0.294
ppm×ppm	1	7.690	3.01%	5.665	5.6651	0.54	0.494
2-Way Interaction	1	4.230	1.66%	4.230	4.2300	0.41	0.452
T×ppm	1	4.230	1.66%	4.230	4.2300	0.41	0.452
Error	5	52.191	20.43%	52.191	10.4382		
Total	13	255.429	100.00%				
Regression Equation in Uncoded Units							
H							
C	K	=	731,080 − 66,446 T + 0.121 ppm + 1510 T×T + 0.000000 ppm×ppm − 0.00558 T×ppm				
IAA	K	=	731,069 − 66,446 T + 0.121 ppm + 1510 T×T + 0.000000 ppm×ppm − 0.00558 T×ppm				
IBA	K	=	731,070 − 66,446 T + 0.121 ppm + 1510 T×T + 0.000000 ppm×ppm − 0.00558 T×ppm				
NAA	K	=	731,066 − 66,446 T + 0.121 ppm + 1510 T×T + 0.000000 ppm×ppm − 0.00558 T×ppm				

**Table 3:** Analysis of variance.

Source	DF	Seq. SS	Contribution	Adj. SS	Adj. MS	F-Value	*p*-Value
Model	8	51.1782	80.37%	51.1782	6.3973	2.56	0.158
Linear	5	15.2312	23.92%	24.4079	4.8816	1.95	0.240
T	1	7.0482	11.07%	0.0039	0.0039	0.00	0.470
ppm	1	1.6829	2.64%	5.4907	5.4907	2.20	0.198
H	3	6.5001	10.21%	9.7321	3.2440	1.30	0.372
Square	2	20.7719	32.62%	9.4434	4.7217	1.89	0.245
T×T	1	20.1617	31.66%	1.7125	1.7125	0.69	0.446
ppm×ppm	1	0.6102	0.96%	2.0630	2.0630	0.83	0.405
2-Way Interaction	1	15.1751	23.83%	15.1751	15.1751	6.07	0.057
T×ppm	1	15.1751	23.83%	15.1751	15.1751	6.07	0.057
Error	5	12.4983	19.63%	12.4983	2.4997		
Total	13	63.6765	100.00%				
Regression Equation in Uncoded Units							
H							
C	RM	=	−253,417 + 22,998 T + 0.2335 ppm − 522 T×T − 0.000000 ppm×ppm − 0.01056 T×ppm				
IAA	RM	=	−253,419 + 22,998 T + 0.2335 ppm − 522 T×T − 0.000000 ppm×ppm − 0.01056 T×ppm				
IBA	RM	=	−253,419 + 22,998 T + 0.2335 ppm − 522 T×T − 0.000000 ppm×ppm − 0.01056 T×ppm				
NAA	RM	=	−253,416 + 22,998 T + 0.2335 ppm − 522 T×T − 0.000000 ppm×ppm − 0.01056 T×ppm				

## Data Availability

The original contributions presented in the study are included in the article. Further inquiries can be directed to the corresponding author.

## Abbreviations

The following abbreviations are used in this manuscript:

CCD	Central Composite Design
df	Degrees of Freedom
H	Hormone Type
IAA	Indole-3-Acetic Acid
IBA	Indole-3-Butyric Acid
K	Callus Formation
NAA	Naphthaleneacetic Acid
ppm	Parts Per Million
R	Rooting Percentage
RM	Root Length
RSM	Response Surface Methodology
SD	Standard Deviation
T	Temperature
